# Low-back electromyography (EMG) data-driven load classification for dynamic lifting tasks

**DOI:** 10.1371/journal.pone.0192938

**Published:** 2018-02-15

**Authors:** Deema Totah, Lauro Ojeda, Daniel D. Johnson, Deanna Gates, Emily Mower Provost, Kira Barton

**Affiliations:** 1 Department of Mechanical Engineering, University of Michigan, Ann Arbor, MI, United States of America; 2 College of Engineering, University of Michigan, Ann Arbor, MI, United States of America; 3 School of Kinesiology, University of Michigan, Ann Arbor, MI, United States of America; 4 Department of Computer Science and Engineering, University of Michigan, Ann Arbor, MI, United States of America; University of Electronic Science and Technology of China, CHINA

## Abstract

**Objective:**

Numerous devices have been designed to support the back during lifting tasks. To improve the utility of such devices, this research explores the use of preparatory muscle activity to classify muscle loading and initiate appropriate device activation. The goal of this study was to determine the earliest time window that enabled accurate load classification during a dynamic lifting task.

**Methods:**

Nine subjects performed thirty symmetrical lifts, split evenly across three weight conditions (no-weight, 10-lbs and 24-lbs), while low-back muscle activity data was collected. Seven descriptive statistics features were extracted from 100 ms windows of data. A multinomial logistic regression (MLR) classifier was trained and tested, employing leave-one subject out cross-validation, to classify lifted load values. Dimensionality reduction was achieved through feature cross-correlation analysis and greedy feedforward selection. The time of full load support by the subject was defined as load-onset.

**Results:**

Regions of highest average classification accuracy started at 200 ms before until 200 ms after load-onset with average accuracies ranging from 80% (±10%) to 81% (±7%). The average recall for each class ranged from 69–92%.

**Conclusion:**

These inter-subject classification results indicate that preparatory muscle activity can be leveraged to identify the intent to lift a weight up to 100 ms prior to load-onset. The high accuracies shown indicate the potential to utilize intent classification for assistive device applications.

**Significance:**

Active assistive devices, e.g. exoskeletons, could prevent back injury by off-loading low-back muscles. Early intent classification allows more time for actuators to respond and integrate seamlessly with the user.

## Introduction

Back pain and injury are highly prevalent, often leading to missed work days and potentially debilitating problems [1, 2]. Lifting belts and passive lumbar supports commonly provide static methods for preventing such injuries in the workplace. Although these braces serve as postural reminders and limit the range of motion, they cannot dynamically off-load the lower back, thus limiting their effectiveness [3, 4].

Recent literature has focused on the development of active assistive devices that have the capacity to dynamically off-load musculoskeletal forces and potentially prevent lower back injury. Existing active back braces consist of large-scale, full-bodied or lower-extremity, braces (exoskeletons). A number of these devices, including BLEEX [5], HULC^™^ [6] and HEXAR [7, 8], augment the user’s ability to lift or carry heavy loads by transferring torques from the torso to the ground through rigid body linkages. While effective in some capacities, these devices do not identify the specific load value being offset by the device, and thus have a limited response capacity for appropriately off-loading the user.

A common limitation in lifting-based exoskeletons stems from the lack of user-based models or classification algorithms embedded within the control architecture to enable predictive assistance [9]. Developers of intelligent exoskeletons such as the Hybrid Assistive Limb (HAL) have successfully integrated controllers that predict the user’s intention to walk by detecting a shift in the center of gravity [10]. However, when it comes to lift assistance or load support, HAL relies on feedback control by providing assistance as a function of measured bioelectric signals. Feedback control relies on reactionary events, initiating a controlled response based on the user’s motion [11–16]. It is not predictive. In many cases, electromyography (EMG) signals are monitored and an assistive torque is initiated once a preset threshold of maximum EMG amplitude has been exceeded. This EMG threshold triggering is used for controlling the HAL exoskeleton to assist a caregiver in a patient lifting task for bathing care [10]. Sato and Yagi (2011) also employ EMG triggering to activate their power-assist suit, which uses pneumatic actuators to aid weighted lifting. To ensure correct torque calculations, the user manually enters the lifted load value into the model-based algorithm. This group also developed an exoskeleton powered by electric motors [16] that requires the user to trigger the lift-assist torque by pressing an instrumented glove. The requirement for manually entered or triggered loads introduces a delay into the system, thus limiting the efficiency and actuation speed of the device. Excessive delays hinder the real-time dynamic response of the device, and result in assistance that may not meet the requirements at a given point in time.

Automated, early, and accurate load detection from user sensor data would address the need for real-time, active assistance. Automated load classification is a form of human intent recognition that requires the use of simple yet robust classification techniques. Early load classification is especially key for providing sufficient response time for the controller to activate the actuator within the device prior to full load support by the user. Identifying user intent (e.g. load to be lifted) in real-time enables the controller to determine the appropriate torque to be applied in a given task and respond quickly enough to assist the user without disruptions in the lifting cycle.

The objective of this work is to quantify the earliest time window at which accurate load classification can be performed. We propose a model-free [9] classification approach for automatically detecting a load to-be-lifted, without the need for musculoskeletal models or manually entered load parameters. We investigate the accuracy of this classification at time windows both prior and post full load support by the user. Optimal time windows providing the highest classification recall are presented for before and after full load support. The classification results include data from nine subjects lifting three different weight classes.

The key contributions of this research include:

- Development of an in-situ classification methodology that enables real-time data analysis and load classification during back lifting tasks. This approach is key to enabling real-time device control.
- Identification of seven descriptive metrics from EMG signals that enable a priori classification of a back lifting task.
- The first demonstration of dynamic task classification for a weight lifting task with >80% accuracy.

This study demonstrates feasibility of real-time task classification of a dynamic task prior to full task onset. These results provide the first step towards smarter active assistance that more effectively off-loads lower back muscles during a dynamic lifting task.

## Methods

Nine healthy subjects (4 female, 5 male; age: 24 (±2) yrs; weight: 148 (±22) lbs; height: 170 (±11) cm), with no history of chronic pain, participated in this study. The study was approved by the University of Michigan’s Institutional Review Board (IRB # HUM00075027) and all subjects provided written informed consent prior to their participation.

**Fig 1:**
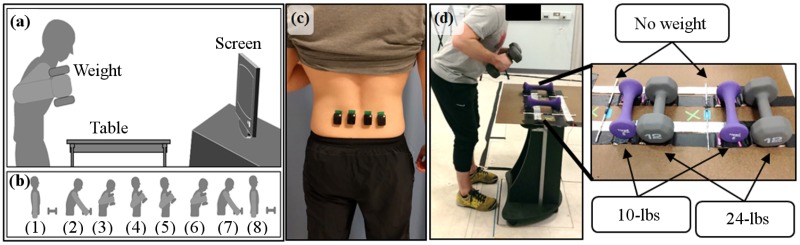
The experimental setup. (a) Schematic showing a subject lifting a weight from a table by following a posture sequence shown on the screen. The table height was adjusted such that trunk flexion was near 30° for each subject. (b) The sequence of posture images shown to the subject on the screen accompanied by a timed sound cue. At the start of each lift, the screen displayed a prompt informing the subject which weight to lift—no-weight, 10-lbs, or 24-lbs, then it displayed the posture sequence with a 1 second delay between each of the numbered (1)-(8) posture images. (c) Low-back muscle activity was measured from four surface EMG bipolar electrodes placed at L4/L5 vertebrae. (d) A subject performing a 24-lbs lift with a close up of the weights on the table. The no-weight case consisted of two sticks wrapped in foil and positioned to close a circuit between two charged foil railings. The force plate under the table can also be seen, flush with the floor, in the larger image.

During all trials, four surface electromyography (EMG) bipolar electrodes (Trigno^™^ Wireless System, Delsys Inc., Natick, MA, USA) recorded activity from the lumbar paraspinal muscles at 1.2 kHz. Electrodes were placed 2 cm apart in a row at the low-back, laterally and medially on the left and right sides of the L4/L5 vertebrae (Fig 1c). The center point, about which the electrodes were symmetrically placed, was located by finding the center of a virtual horizontal line along each subject’s back connecting the tops of the left and right superior iliac crests [17]. The major muscle groups in this region are the multifidus and erector spinae muscles [18, 19].

Each subject participated in a single testing session, involving a maximum effort task and a lifting protocol. After a short warm up, subjects performed two maximal voluntary isometric back extensions on a roman chair [20]. The maximum of those two measures represented that participant’s maximum voluntary contraction (MVC). Next, subjects repetitively lifted pairs of weights from a table by following a specific, timed posture sequence (Fig 1a and 1b). Participants lifted three loads: 1) two very light sticks (‘no-weight’), 2) two 5-lbs dumbbells (‘10-lbs’), and 3) two 12-lbs dumbbells (‘24-lbs’) 10 times each (30 total lifts per subject). The order of the lifted weights was randomized. Each complete lift was followed by a 5-second rest period. Subjects learned to follow the posture prompts during a training lifting session prior to data collection.

The weights lifted during the lifting protocol rested on a table positioned on a force plate (AMTI Inc., Watertown, MA, USA). The table height was set such that each subject had a forward trunk flexion angle of about 30° when reaching the weights. The force plate signal detected when the 10-lbs and 24-lbs loads were lifted. A circuit setup detected the lifting of the sticks: the sticks were wrapped in foil and placed on top of two charged foil rails (Fig 1d). Lifting the sticks opened the circuit between the rails and recorded a voltage drop.

**Fig 2:**
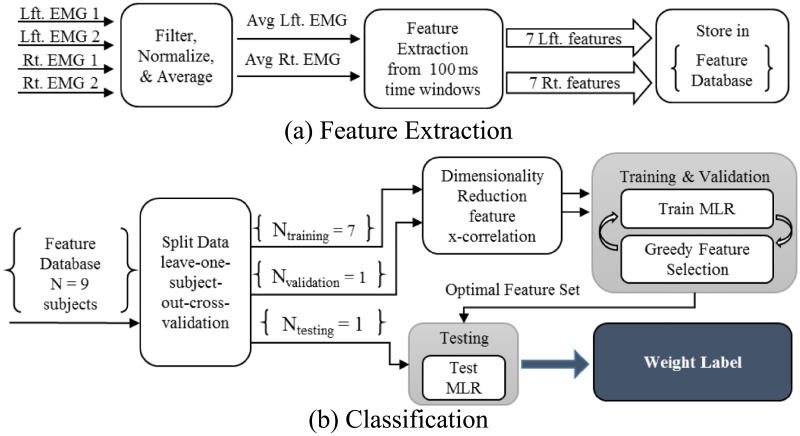
Data analysis steps. Flowcharts showing the steps involved in (a) data processing, segmentation and feature extraction, and (b) dimensionality reduction, feature selection, cross-validation and, finally, testing.

Data analyses are outlined in Fig 2. The following subsections detail the pre-processing data analysis steps, classifier input preparation and the classification process. All analyses were performed using MATLAB and the Statistics and Machine Learning Toolbox Release 2015b (The MathWorks Inc., Natick, MA, USA).

### Data filtering and normalization

EMG data from the four surface electrode channels were bandpass filtered (20 Hz to 300 Hz), band stop filtered (59 Hz to 61 Hz) to remove electrical noise, de-meaned, full-wave rectified, and low-pass filtered (4 Hz) to obtain a linear envelope [21]. All the filter designs included 5th order Butterworth filters with zero-phase filtering. Dividing the linear envelope by the maximum EMG, obtained from the maximum effort task, normalized the data to a percentage of the maximum voluntary contraction (% MVC) for each subject. The % MVC data for the two left and two right EMG channels were averaged to produce two signals (average left and average right) after a cross-correlation analysis showed high correlations (> 0.96) for all channel combinations. One of the subjects had a faulty right medial EMG channel, which was excluded in the analysis and the right lateral channel was used instead of the average right activity for this subject. To improve classification and account for inter-subject strength differences, the averaged % MVC data was further normalized by dividing all lifts for a single subject by the maximum % MVC observed in that subject’s first 24-lbs lift.

A threshold detection algorithm found the time at which each load was completely lifted off the table and, thus, fully-supported by the subject. This time point was termed **load-onset**. Threshold detection of the vertical-axis raw force plate signal and of the sticks signal (connected to the foil rails) identified load-onset for the weighted (10-lbs and 24-lbs) and no-weight lifts, respectively. All lifts for all subjects were aligned according to the time of load-onset, defined as time zero. The regions of interest spanned 2 seconds before (**pre-onset**) and after (**post-onset**) the load-onset time point.

### Segmentation and feature extraction

Time series average left and right % MVC data from each lift were segmented into 100 ms segments overlapping by 50 ms. Other studies have employed varying time window lengths ranging from 32 ms [22] to 500 ms [23], with varying levels of overlap. The 100 ms window was chosen as a trade-off between having sufficient data for feature extraction and a small enough window to allow enough time for signal classification and implementation of an assistive action within a lift. Subjects completed a single full lift cycle in about 8 seconds.

Seven time domain descriptive statistics features were extracted from each 100 ms time window segment: mean, standard deviation (SD), difference (diff), maximum (max), minimum (min), amplitude (amp), and root-mean-square (RMS). The diff feature was calculated by subtracting the first data point from the last data point within the time window. Amp was calculated by subtracting the minimum data point from the maximum data point of the samples in the time window segment.

A leave-one-subject-out method divided the nine subjects into training, validation and testing sets, such that each subject was in the testing set once and the remaining subjects were split into validation and training sets using leave-one-subject-out-cross-validation. All features in all sets were z-score normalized to the mean and standard deviation of the pooled values of each feature from the training and validation sets, as Eq 1 illustrates.

$$\begin{array}{c} \text{z-score}\left(y_{n}\right)=\frac{y_{n}-mean\left(\left\{Y_{train},Y_{val}\right\}\right)}{SD(\left\{Y_{train},Y_{val}\right\})} \end{array}$$ (1)

In Eq 1, *y*_*n*_ represents the value of a feature at time window segment *n*, and {*Y*_*train*_, *Y*_*val*_} is the set of pooled values of that feature across all time window segments for the data in the training and validation sets.

### Dimensionality reduction and classification

To avoid singular matrices in the classifier, redundant features must be identified and removed. Cross-correlation of all fourteen features (seven features from each averaged left and right EMG channel) identified redundant features, with features scoring a correlation value of 0.80 or higher removed from the data. Classifier input used only non-redundant features in the training set. Next, a multinomial logistic regression (MLR) classifier was trained using features of training lifts and their known weight class labels (i.e. ‘no-weight’, ‘10-lbs’ and ‘24-lbs’). A greedy feedforward feature selection method added features one at a time to maximize classification accuracy of the validation feature set, resulting in an iteratively identified optimal set of input features. The feature selection process ended either when a) all the possible features had been selected, or b) the validation classification accuracy could no longer be improved by adding any of the remaining features to the optimal feature set.

The training and validation process generated an optimal feature set for each time window segment. The optimal features were then extracted from the test set and classified with a MLR function that had been re-trained with the pooled optimal training and validation features. For each time window segment, the MLR classifier outputted three label probabilities of the segment belonging to a lift from each of the three classes: ‘no-weight’, ‘10-lbs’, or ‘24-lbs’. The class with the maximum probability was taken as the output label for that lift at that time segment. A comparison of the output and the true class labels indicated classifier performance through two metrics: classification *accuracy* and *recall*. Accuracy is the percentage of correctly classified lifts, and recall is the percentage of correctly classified lifts within each weight class.

The MLR classifier was chosen over other methods for its simplicity and fast processing time. Moreover, MLR is a probabilistic classifier, which is beneficial when considering classification of more than two classes.

### Statistical analysis

A repeated-measures ANOVA, constructed using Minitab^®^16 Statistical Software (Minitab Inc., State College, PA, USA), investigated whether differences in recall values, at the time windows with the highest classification accuracy, were significant between the different weight classes. The ANOVA investigated effects from the following factors and their interactions: the weight class label, onset time frame (pre- or post-onset) and subject number. The subject number was set as a random factor. A p-value lower than 0.05 was considered significant.

## Results

**Fig 3:**
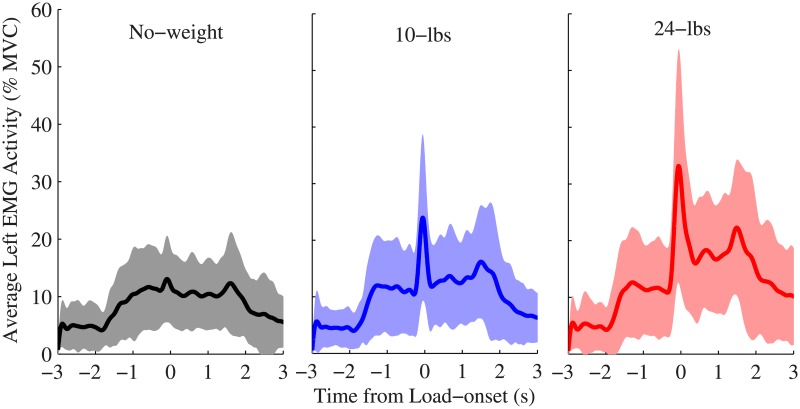
Average muscle activity for different loading conditions. The average muscle activity of the two left EMG channels for all nine subjects normalized to each subject’s maximum voluntary contraction (MVC) baseline. Time zero is the time of load-onset. The shaded regions show the region of ±1 standard deviation around the average. There is a clear spike in average activity around the load-onset time point that is more prominent with increasing lifted load values. The average of the right EMG channels showed similar activity patterns.

**Fig 4:**
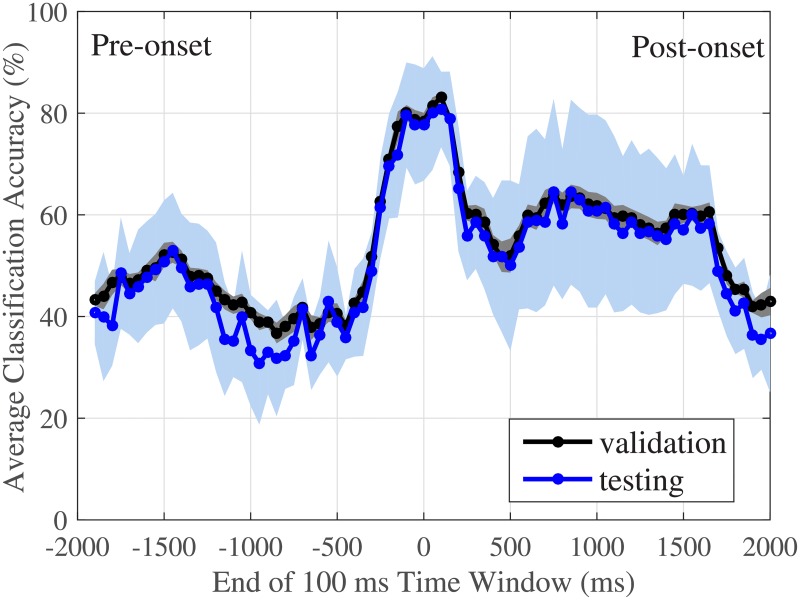
Classification accuracy increases at time windows near load-onset. The classification accuracy from the testing and the cross-validation steps for each 100 ms time window segment. The plot shows average accuracy from nine subjects. The shaded regions illustrate ± 1 standard deviation at each time point. The testing curve matches the validation curve, indicating that overfitting was avoided. Time zero indicates the time of load-onset.

During the course of a lift, muscle activity increased as subjects bent forward to pick up the weights. The EMG signals indicated a spike in activity just before load-onset as subjects prepared to lift the weight off the table. As shown in Fig 3, the amplitude of the spike increased as the lifted load increased, with the ‘no-weight’ lifts showing significantly smaller spikes than the other two load categories. The data around the spike, i.e. near *load-onset*, resulted in the highest classification accuracy. The region with the highest average testing classification performance (see Fig 4) ranged from 200 ms before load-onset to 200 ms after load-onset, with average classification accuracy ranging from 80% (±10%) to 81% (±7%).

There was very good agreement (cross-correlation of 0.999) between the testing and the cross-validation classification accuracy (Fig 4), indicating that the inter-subject classifier was able to generalize well for this healthy subject population, where participants had varying levels of physical fitness. The highest testing accuracy was observed at two 100 ms time windows: [−200, −100] ms and [100, 200] ms from load-onset. The first window, ending at −100 ms, was the earliest the classifier could predict the load with a greater than 80% average accuracy prior to load-onset. The trained MLR classifier could classify thirty 100-ms segments from 30 lifts in less than 1 ms. Thus, if implemented online, a prediction of the load can be generated in well under 1 ms, using data collected 100 ms *prior* to load-onset.

**Fig 5:**
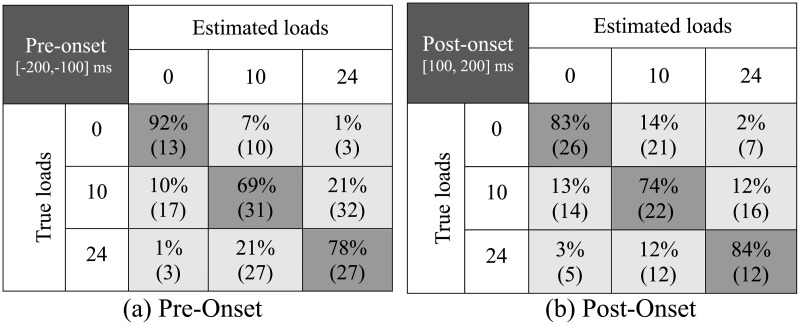
Distribution of classification results. Confusion matrices showing the average classification results (with standard deviations in parenthesis) for the time windows with the highest accuracy during the regions (a) before and (b) after load-onset or full load support.

The statistical analysis, performed for the recall values at the optimal time windows (Fig 5), showed that the only statistically significant factor was the interaction between subject and class (*p* = 0.003). None of the other factors (subject, class, and onset time frame) nor their interactions were statistically significant. Differences in recall between pre-onset and post-onset optimal time windows were not significant (*p* = 0.700), nor was the interaction of class and onset time frame (*p* = 0.296).

**Fig 6:**
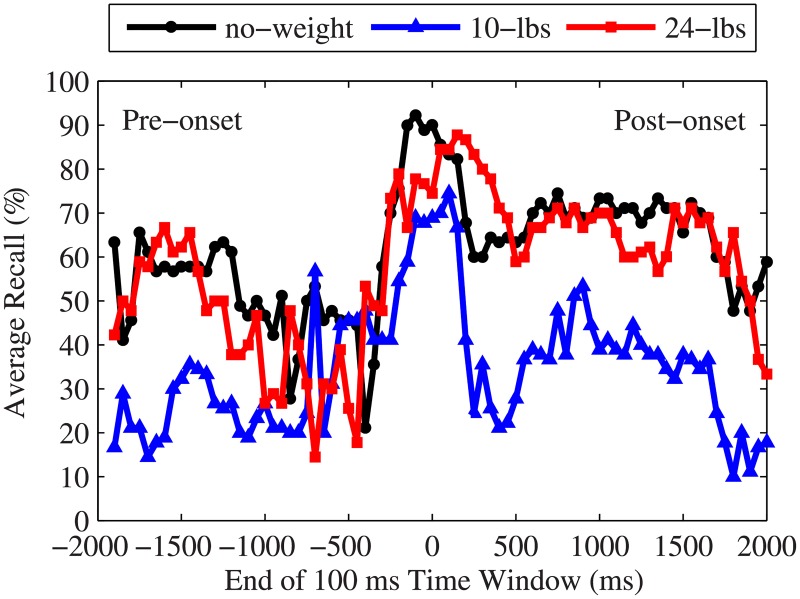
Average classification recall for each weight class. The average testing recall for each weight class at each time window for all nine test subjects. Time zero indicates the time of load-onset.

The average recall for the 10-lbs class consistently fell below the recall for the no-weight and 24-lbs classes (Fig 6). However, at the points of interest, where the highest accuracy is observed, this difference was not statistically significant (*p* = 0.362). Confusion matrices (Fig 5) at the two time segments with highest accuracy (one before and one after load-onset) show that an average of 21% of the 10-lbs lifts were misclassified as 24-lbs lifts prior to load-onset (Pre-onset). Similarly, an average of 21% of the 24-lbs lifts were misclassified as the light weight. This is compared to 10% and 1% of the 10-lbs and 24-lbs lifts, respectively, misclassified as no-weight lifts. However, during the Post-onset time frame, the misclassified 10-lbs lifts were roughly equally split between the no-weight and 24-lbs class labels.

**Fig 7:**
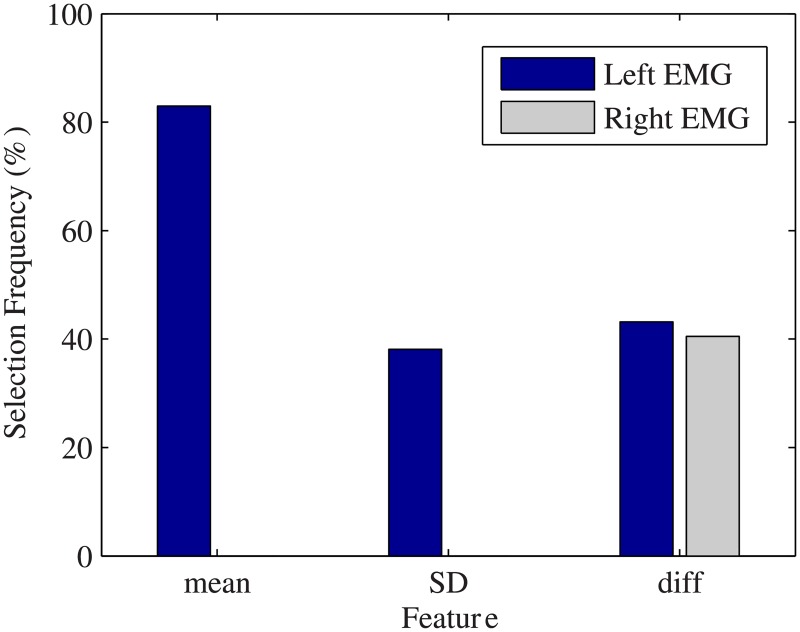
Selected features and their selection frequency. The percentage of times (out of 711) that each feature from each channel was selected for the optimal feature set as input by the greedy feedforward algorithm during cross-validation of the MLR classifier. The mean feature was selected much more frequently than the others.

As for feature selection, the correlation analysis step removed min, max, amp and RMS features because they were found to have correlations of 0.80 or higher with one or more of the remaining features: mean, SD and diff. Furthermore, the average right EMG channel mean and SD were highly correlated with the left channel and removed. Thus, the mean and SD from the left channel and the diff of both left and right channels were the remaining features. In other words, three features remained from the left and one from the right average EMG channel, for a total of 4 remaining features. The greedy feedforward feature selection generated a total of 711 (79 time window segments x 9 test subjects) optimal feature sets from the remaining features. A majority of the 711 sets (83%) contained the mean, making it the most frequently selected feature. The remaining features, the SD from the left channel and the diff from right and left channels, were all selected in similar frequencies with 38% to 43% of the 711 sets containing them (see Fig 7). The optimal sets containing only 2 features made up 47% of the 711 sets, and 26%, 23%, and 3% of sets included 1, 3, and all 4 features respectively.

## Discussion

The earliest window when loads can be classified with 80% accuracy was −200 to −100 ms prior to load-onset, which corresponds with the electromechanical delay of the low-back erector spinae muscles at the L5 region [24]. This classification performance during pre-onset was not statistically significantly different from post-onset performance. Thus, it can be argued that loads can be classified 100 ms prior to full load support with as high of an accuracy as after load-onset. As mentioned in the Introduction, the earlier a load classification can be made, the more time a controller and actuator have to respond. Once the load is classified, a control command must be generated and sent to an actuator, which will provide the appropriate assistive torque to off-load the user’s muscles. With load classification completed 100 ms prior to load-onset, an actuator with a 100 ms or less response time would ensure that an assistive torque can be initiated prior to full load support by the user. A few common actuator response times are listed in Table 1, ranging from 37.5 to 315 ms. The 100 ms head start afforded by the early classification shown in this study gives us a significant advantage over manual force input or post-onset detection methods, which indicates greater potential for device actuation prior to significant user loading. The use of preparatory muscle activity pattern recognition brings us a step closer towards seamless integration of device and user for effective injury prevention.

**Table 1:** A summary of relevant response times of muscles, classifiers, controllers and actuators commonly used in assistive device applications.

Type of Action	Action	Time Taken	Device/Muscle Group	Ref.
Muscle Delay	electromechanical delay	131–147 ms	Erector Spinae muscles at L5 vertebrae	[24]
	electromechanical delay	110–140 ms	wrist flexor muscles	[25]
Command Generation	classify EMG and generate control command	250 ms	wrist exoskeleton (WAP)	[26]
Actuator Response	torque control bandwidth	314 ms	WREX	[27]
	force control bandwidth	50 ms	Pneu-WREX	[28]
Full Control Loop	control loop period	37.5 ms	power-assist suit for aiding weighted lifting	[15]

WREX is an upper limb exoskeleton with series elastic actuators; Pnue-WREX is an upper limb exoskeleton with pneumatic actuators.

During pre-onset, the classifier confused the two weighted lifts (10-lbs and 24-lbs) with each other, labeling 21% of the 24-lbs lifts as 10-lbs and 21% of the 10-lbs lifts as 24-lbs compared with only 10% as no-weight lifts. However, during post-onset, 13% of the 10-lbs lifts were misclassified as no-weight and 12% as 24-lbs. This indicates that subjects might exhibit similar preparatory activity when expecting to lift a weight regardless of value, but once the lifting begins, activity is adjusted and lifts requiring less applied torque from the user may be confused with no-weight lifts.

**Fig 8:**
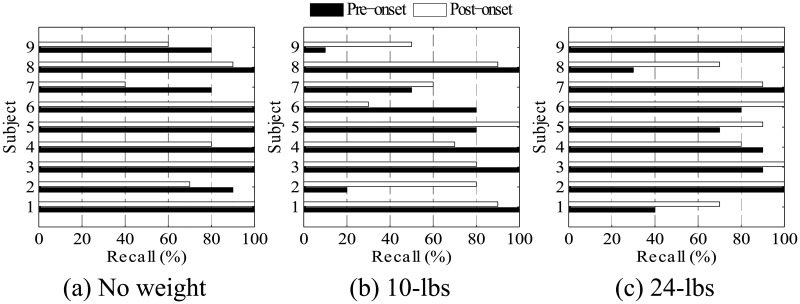
Classification recall was affected by subject and weight class. Classification recall for each subject during the optimal time windows before (Pre) and after (Post) load-onset. The interaction between subject and weight class had statistically significant affects on recall percentage.

No statistically significant differences in recall between the different weight classes were observed during the pre- and post-onset optimal time windows. Interaction of weight class and subject, however, was statistically significant (*p* = 0.003). This indicates that some subjects were difficult to classify for certain lifted weights but had high recall for other weight classes (see Fig 8). The classifier was tested with data from one subject at a time and trained using data from a different set of subjects. There was no cross-over between subjects in the test set and subjects in the training set. While, this may make it difficult to predict the performance of this classifier for new subjects, the implementation of cross-validation with an isolated validation subject set different from the training set did aid in increasing overall classification performance. Intra-subject EMG pattern-recognition (e.g. [29], [26]), where data from the same single subject is used for training and testing, is more common than inter-subject modeling, especially for impaired populations. However, this requires a sufficiently large data set to be obtained from each subject for training, validation and testing, which can be a time consuming and daunting process involving repetitive data collection trials. Combining data from several subjects provides a larger set of training observations for the classifier with less repetitions for each subject. The downside remains to be a likely decrease in classification accuracy, as the classifier attempts to generalize to multiple subjects.

The classifier presented here used descriptive statistics of the linear envelope of the signal. The linear envelope filtering maintains important amplitude information for load detection while significantly decreasing the noise. There are a number of other feature sets in the time and frequency domains using raw EMG signals, time windows, and EMG channel combinations that could be explored [29, 30] to potentially improve classification. However, this work was a feasibility study of the potential for EMG-based early load classification during dynamic lifting and not a feature selection investigation. A wide array of other classifiers could also be used, such as linear discriminant analysis (LDA) [29–31], fuzzy-logic [22], neural networks [32, 33], hidden Markov models [34], or Bayesian models [35]. The advantage of the probabilistic MLR classifier for this pilot study was simplicity and fast processing time, in addition to its reduced sensitivity to outliers compared to other classifiers such as LDA.

The challenge remains in knowing when to trust the classifier for on-line control, i.e. “How do we know we are looking at an optimal time window in real-time?”. The answer to this question lies in probabilistic modeling and sensor fusion to identify the lift cycle and associate confidence values with classifier predictions at different times within the lift cycle. Sensor fusion has been shown to improve classification in similar applications for lower-extremity assistive devices [35, 36]. A limitation of this work is that the lifting pace was controlled through subject training and instruction and the EMG patterns could change with varying lifting speeds [37]. Sensor fusion and Bayesian models could further improve the accuracy and account for varying lifting speeds. Moreover, the use of high-density EMG electrodes [38] or placing electrodes on other muscles that aid lifting (such as the leg muscles) could further improve the force estimation accuracy. However, the additional signals would require additional processing, thus increasing processing time, and cross-talk could add further noise to the data. Another potential limitation in this approach is that fatigue could influence the EMG readings during the lifting task. Importantly, the range of weights used (0 to 24-lbs) along with controlled slow movement speeds, and rests incorporated in the protocol helped to mitigate fatigue affects. An analysis of the maximum observed EMG within a lift showed very small variations (±5% MVC) between lifts for each subject and weight class. This indicates that fatigue effects were minimal.

## Conclusion

This study found that an EMG signal can be used to classify lifted loads, as early as 100 ms prior to load-onset, i.e. when the load is fully supported by the subject. This finding encourages the use of myoelectric-based control strategies for assistive devices that aid weighted lifting. The results demonstrate that early load classification is possible, which will allow the controller more time to respond and meet optimal controller delay specifications for seamless integration of intent-recognition and actuated assistance. The classification generalized well among the healthy subject population, achieving average accuracies greater than 80% using inter-subject cross-validation and testing. Nevertheless, the generalizability of the results is limited by the small sample size and lack of diversity of the subject pool. Future work should include a more heterogeneous participant group (in terms of age and health/fitness level) to allow further extension of the results. Moreover, this initial study demonstrated the ability to classify only three discrete loads in the 0 to 24-lbs range. We expect these findings will extend toward classification of other loads, particularly as larger changes in loads might be more readily identifiable as differences in EMG patterns are expected to be more pronounced. Future work should include a more thorough exploration of optimal features, time windows, and classification schemes to ensure optimal classification accuracy, greater granularity in loading, as well as employing sensor fusion and Bayesian modeling. The classifier algorithm can be implemented online and has the potential for incorporation into real-time control of active assistive devices.

## Supporting information

S1 File Source data. This file archive contains the linear envelopes of the average left and right EMG data from each lift used to generate the results in this paper. All nine subjects and three loading conditions are included, along with the sex, age, height and weight of each subject.
(ZIP)
